# American Dental Association

**Published:** 1890-09

**Authors:** 


### Reports of Society Meetings.

### AMERICAN DENTAL ASSOCIATION.—THIRTIETH ANNUAL MEETING, EXCELSIOR SPRINGS, MO., AUGUST 4 TO 8, 1890.

*Tuesday Morning's Session.*

THE Thirtieth Annual Meeting of the American Dental Association was held at Excelsior Springs, Mo., August 4 to 8. The convention was called to order at eleven o'clock Tuesday morning, Dr. M. W. Foster, of Baltimore, Md., presiding. The sessions of the convention were held in the Elms Opera-House. Other officers present were Dr. A. W. Harlan, of Chicago, First Vice-President; Dr. J. D. Patterson, of Kansas City, Mo., Second Vice-President; Dr. George H. Cushing, of Chicago, Recording Secretary; and Dr. A. H. Fuller, of St. Louis, Mo., Treasurer.

After the call of the roll and the adoption of a resolution dispensing with the reading of the minutes, Dr. Gardiner, of Chicago, offered the following preamble and resolution:

"WHEREAS, There is to be a World's Columbian Exposition in Chicago in 1893; and,

"WHEREAS, In consequence of the fact that the choicest products of the world are to be there displayed, it is expected that citizens in large numbers of all civilized countries will be gathered together there for the purpose of seeing these exhibits; and,

"WHEREAS, The time of the Exposition will be an opportunity for a great meeting of the dentists of the world; and,

"WHEREAS, It is believed that a great advance in science and the practice of dental and oral surgery would result from a meeting of the dentists of the United States with those from foreign countries who might then be visiting this country; and,

"WHEREAS, It is desirable that any meeting then held should be at the in stance of the American Dental Association and the Southern Dental Association, and organized by a joint committee by them appointed; therefore, be it

"*Resolved*, That the president of this association appoint a committee of five to confer with a like committee, appointed by the Southern Dental Association at its last meeting, upon this subject, and that this joint committee have the power to fill all vacancies, and shall add to its membership either one, three, or five more members, as it may deem advisable; and when this committee is so completed it shall be clothed with full power to take such action as it in its judgment may deem best for creating an organization for the purpose of holding a dental meeting in Chicago in 1893, which the reputable dentists throughout the world shall be invited to attend, and that any action that this committee may take in the premises shall be final and binding."

Dr. Alport, of Chicago, denounced this as a discourtesy to the Southern Dental Association, whose committee was then on the floor awaiting an opportunity to submit a similar resolution. Dr. Alport favored co-operating with the Southern Association in this matter, and he would not consent to this method of procedure. He moved to lay the resolution on the table to give the committee referred to an opportunity to perform its mission. Dr. J. H. Thompson, of Topeka, Kan., moved the adoption of the resolution. Dr. J. A. Swazey, of Chicago, moved a substitute. Dr. James Truman, of Philadelphia, moved a reference of the whole subject to a committee of three, with instructions to report. Dr. Stockton, of New Jersey, wanted the matter settled without delay. Dr. Rhein, of New York, objected to the plenary powers proposed to be conferred on the committee by the resolution, and favored the reference of the subject to a committee, with instructions to report. A motion ordering the previous question in order to close the debate was voted down. Dr. Cushing, of Chicago, offered a resolution to table the whole matter temporarily in order to afford the committee of the Southern Association the opportunity to present the resolutions adopted by that body which are in substance about the same as that offered by Dr. Gardiner. Later in the morning session, Dr. Carpenter, of Atlanta, Ga., chairman of the committee appointed by the Southern Dental Association, was called upon and submitted the resolutions of that body. The resolutions were read by the secretary, Dr. Cushing, and unanimously endorsed, except that Dr. Rhein, of New York, still adhered to his objections to the absolute powers to be granted to the committee. The president was instructed to appoint a committee of five from the American Dental Association to co-operate with the committee from the Southern Association.

A communication from the S. S. White Manufacturing Company of Philadelphia created considerable surprise. The company has been printing the reports of proceedings in the *Dental Cosmos.* Last year Dr. W. A. Barrett, of Buffalo, in discussing dental education, attacked the S. S. White Manufacturing Company and denounced in bitter terms the partnership carried on by the society. Dr. J. N. Crouse, of Chicago, introduced a resolution expressing confidence in the S. S. White Company so far as the publication of the reports is concerned, and denying that the society shared the views of the member from New York State. Dr. J. R. R. Patrick, of Belleview, Ill., thought that the society as a scientific body ought to be self-reliant, and insisted that it was perfectly able to publish its own reports. After a spirited discussion the communication, which was a lengthy one, dealing with the history of the arrangement under which publication of the transactions is made and pointing out the benefits that the association derives from the contract, was referred to a committee consisting of Drs. W. X. Sudduth, W. H. Smith, and George S. Cushing.

President Foster then delivered his address, Vice-President Harlan being in the chair. The points of greatest interest to the dentists of the country are given. After a brief and formal introduction President Foster said,—

Among the many subjects to be presented it has occurred to me that it would be well to formulate a plan of action in regard to making the State dental laws uniform.

I would suggest that this body take into serious consideration the advisability of a uniform set of laws regulating the practice of dentistry in all of the States. It is perfectly obvious that we are dealing here with a subject of much importance and upon which a great variety of opinion exists. The rights, the best interests of our profession, the colleges, and the newly-graduated students are all involved. The public welfare, as well as the interests of our profession, demands such a protection as will eventually destroy all ignorant charlatanry and free the people from malpractice and indifferent treatment. We are all aware of this. We are all in favor of it. Colleges and young graduates alike demand our protection.

We must admit that our colleges and associations have made dentistry what it is to-day. As in the past so in the future. We must look to those institutions as our best friends and auxiliaries to aid and to push forward the work that is to be accomplished.

From a hasty analysis of our State statutes we find that our laws regarding the practice of dentistry in the different States are by no means uniform in their requirements. This is not as it should be, and if continued will bring on a conflict between the associations of the several States which will weaken if not destroy our national existence as a dental profession.

1. We have laws which demand that both graduates and non-graduates shall be examined by State boards before admission to practice.

2. We have laws recognizing the holders of the certificates of reputable schools. These permit the holder to practise to the exclusion of all others desiring this privilege.

3. We have laws demanding examinations, but allowing this favor to graduates only.

4. We have laws demanding the examination of dentists whether graduates or not, with exceptional clauses in favor of the holders of the medical degree.

In regard to the first mentioned,—that is, laws which examine all alike, whether graduates or not,—it would seem at first glance without serious objection. It seems fair to judge an applicant for the privilege of practice by what he knows. But is it fair to place a man who has no college training on the same platform with one who has enjoyed at much expense of time and money this advantage? No medical board that I know of permits this, and if these laws were universally adopted, would it not effect seriously the interests of our colleges? What would the members of our profession who have so earnestly advocated the extension of college preparation and training have to say to this, particularly when the colleges in obedience to their suggestions have already lengthened their time of preparation from one to three sessions?

Let us look a moment at the workings of such a law. I know that in one of our States a would-be student of dentistry was advised, as his means were limited, that it would be much better for him to prepare himself at home and go before a board of examiners and thereby save himself much time and money, since at last, if he should go to college and graduate, he would be subjected to the same ordeal. This looks like progressing backward.

In another State, the laws of the State dental association require that a member shall take, as pupils to prepare for college, only those who will remain under the preceptorship two years. This added to the three years required by the college would aggregate five years of preparation prior to admission to practice. Under such circumstances, do you not believe that the average man would decide to prepare himself at home and let the college go? These State laws make no discrimination in regard to previous preparation. If the applicant is twenty-one years of age and can pass the examination, he is permitted to practise.

Again, does not this forced examination of college graduates show to the whole world an implied distrust and want of confidence in our own schools? Even foreign countries recognize some of our degrees. Is not this a national degradation? And is it not calculated to injure and destroy our own colleges.

Second, we have laws which recognize the diplomas of all reputable schools and allow only such the privilege of practice. It seems to us that laws like these evidently take the ground that a college training is imperatively necessary to a practitioner, and forbid all other consideration. They do not suppose it even possible that a practitioner can be qualified for practice in the office of a practitioner.

Third, laws which demand examinations for all graduates, but exclude all others from this privilege. Laws like the last quoted concede, too, that the graduate alone is competent and fit to practise. In this way they aid and encourage the colleges, but they throw a wet blanket on them by the publication to the world that we have not a single college in the United States, like Cæsar's wife, above suspicion, no dental diploma which can be trusted and relied upon, thereby destroying the reputation, the prestige, and the value of every degree in our country. It does seem proper that the student who has spent so much time and money in procuring his degree should have some protection and exemption from the torture of further examinations. Under these laws there is no respite or relief for him; no matter where he settles, this ordeal awaits him; if he passes the examination in one State, and for reason emigrates to another, the same ordeal of torture is inflicted. I understand perfectly that the response from those favoring the laws, in reply to what I have said, will be that if the college graduate is not tested by such examinations the colleges would be under no restraint and would cease possibly to be so exacting in their requirements, and make, in a great many instances, improper graduations. There is much reason in this. But is a State board any more immaculate in its decisions than are the college Faculties? Is the State board any less human? Is it more competent? Is it always appointed because of competency and integrity?

And is there not some way by which this matter can be disposed of alike just to all concerned and interested? I respectfully urge that this question be discussed and carefully considered. We ought from the general intelligence of this Association to have a plan suggested and adopted that will insure the public, the profession, the colleges, and their graduates justice and reasonable satisfaction. We can then announce to the world that our college degrees, both at home and abroad, deserve the confidence of all.

Further, suppose that the State boards in the States where a dental college or colleges exist be required with their Faculties to conjointly examine thoroughly, both practically and theoretically, all candidates for graduation, and when a student has been subjected to such examination and found worthy and deserving, let the diploma be signed by the president and secretary of the board, together with the Faculty, and let such diploma be accepted in every State of the country. Would there be any reason for one State board rejecting the work of another? The public and the profession, through the selection of the members of such boards, ought to be satisfied. The colleges would, I think, rejoice in being relieved of the responsibility and unpleasant duty of turning down incompetent men, and I believe the students would be satisfied. They do not so much dislike examinations as they do the repetition of the examinations. Dr. Riggs used to say that dentists were like bumble-bees, always biggest when first hatched, and they felt a sense of mortification to be obliged to submit to an appearance before a board of examiners.

President Foster also suggested the propriety of petitioning Congress at this time to appoint from the dental profession representatives to the army and the navy of the United States with suitable rank and pay. He said, Congress at this time appears to be in favor of building such a navy as will properly represent this great country. Does any gentleman think that in these progressive days a ship, with her crew going perhaps on a long cruise, effectively manned and officered, is fully equipped without a representative of our profession on board? Is it not inhumanity to man to send a ship's crew off in such a condition, where nothing is left for the poor unfortunate better than to be turned over to the tender mercies of the surgeon, who knows nothing of dentistry beyond the extraction of teeth, and where no one in case of a fractured jaw is capable of applying the interdental splint to relieve the sufferer? We think at this time, especially, that the public would enthusiastically endorse such action on the part of Congress. I need not dwell upon this subject. You all recognize the necessity for action in this matter, and the times are, we think, ripe for it. Of course in this, as in every other effort to effect uniform legislation in the States regarding the practice of dentistry, we should invite the aid and co-operation of the Southern Dental Association and all State Faculties.

I suggest also for your consideration the propriety of endorsing and recommending to the proper authorities the use of models taken from impressions of the mouth in the identification of criminals. I think this would be perhaps a very reliable means of identification, and those models might furnish a very valuable source of study to the ethnologist and physiognomist. In Paris, I am told, they take impressions of the thumbs, and have found this quite a useful method of identification. You are well aware that but for a set of artificial teeth the remains of the murdered Dr. Parkman would not have been identified. You may also be informed that in one of the most notable murder cases on record, in which an attempt was made to defraud the insurance companies out of a large amount of money, the identification of the remains was by the teeth. A skull was presented in court represented to be the skull of the missing man, who was at that time still alive. It was proved that the supposed deceased had an excellent set of teeth. The skull showed the loss of the anterior teeth and absorption of the sockets. Complications arose making it necessary for those in the plot to murder the man to escape imprisonment themselves. This was accomplished, and, having the experience of the former trial in view, an effort was made to knock out the anterior teeth, which was partially successful, but the roots and portions remaining showed that it had been of recent occurrence. This, with the remaining teeth, was the positive means of the identification of the decomposed body.

President Foster recommended the establishment of a head-quarters, dental museum, and library of the Association in the capital of the country. There the archives can be kept safely. Such a museum would grow very rapidly, and in time become very valuable, and redound much to the credit of the Association. We could by the appointment and election of members resident in Washington have always at the head-quarters a permanent officer, who could take care of the museum and library and represent us to foreigners. Our officers are constantly changing, as well as the place of meeting, and a person in a foreign land desiring correspondence with this body would always know where and to whom to address his communications. I feel confident that the government, if properly approached, would willingly aid in this work,—perhaps furnishing rooms in the Smithsonian Institution. The medical department could give us great assistance, and we would soon have at little expense to us something of which we might all feel proud. Probably, too, after a short time the government would offer us, through this channel, great advantages in the study of ethnology and microscopy. There are a large number of possibilities depending on this movement, and I would advise the appointment of a committee to examine into the probable value and success of such an undertaking, and, if favorably considered, to report at the next meeting a plan for carrying it into effect.

The question of a preparatory dental education has been discussed in the meetings of our associations, both orally and in writing, by leading men in the profession, and yet it is not solved. As the better method many advocate a higher education than is now required for admission to the schools. This prevents men of natural capacity from becoming members of our profession. There are many who consider a good English education sufficient. The colleges have agreed to an increase in the length of the sessions, and have endeavored to meet the demand for the more thorough preparation of students before granting a diploma. Does this increase in time bring about the desired reform? Are not some men by educational advantages and manual dexterity more competent and better fitted to practise our profession than others with less qualifications? Is there not a great truth in the language of Professor Huxley,—"Handicap by time one man to the capacity of the other." The broader and more liberal the general education the more liberal and broad are the views of the possessor, and from this stand-point such a one might reflect more credit on our profession than one of less accomplishments. The natural endowments and aptitude of the less educated man in a final examination, would prove that other qualities fit him also for our profession. From this stand-point a dental education commences when the person commences the study of dentistry in the office of a preceptor, or in some reputable school. This general education either qualifies him for this, or it does not. This is plain, I think. Then it is not a question of qualification at all, whether it be one or five years or original capacity.

In closing, President Foster complimented the Association upon the work of the past and the prospects for the future. He spoke of the bright lights of dentistry called from labor to reward, and urged that a scientific body is not the place for selfish ambition or personal aggrandizement. He held that the absorbing desire of all should be to rank dental science among the benefactions of mankind.

(To be continued.)

